# Adherence to Prescribing Guideline-Directed Medical Therapy at Hospital Discharge in Subjects With Acute Coronary Syndrome, and the Relationship With Mortality

**DOI:** 10.7759/cureus.24000

**Published:** 2022-04-10

**Authors:** Hadeel Alkofide, Raghad Alshuhayb, Nibras Alhazmi, Razan Almofada, Asmaa Bin Hazzaa, Amjad Alsharif, Hanan Abouzaid

**Affiliations:** 1 Clinical Pharmacy, King Saud University, Riyadh, SAU; 2 College of Pharmacy, King Saud University, Riyadh, SAU

**Keywords:** st-elevation myocardial infarction (stemi), adherence to preventive measures, mortality, guideline directed medical therapy, acute coronary syndrome

## Abstract

Introduction

The use of guideline-directed medical therapy (GDMT) after acute coronary syndrome (ACS) is associated with a significant reduction in mortality; however, suboptimal prescribing of these therapies has been reported. This study aims to determine adherence to prescribing GDMT in subjects with ACS at hospital discharge and to measure the relationship between this adherence and one-year mortality.

Methods

A retrospective cohort study was conducted on adults admitted with an ACS. The primary outcome was adherence to GDMT, defined as compliance with prescribing aspirin, angiotensin-converting enzyme inhibitors (ACEIs), or angiotensin receptor blockers (ARBs), beta-blockers, and high-intensity statins, according to international guideline recommendations. The secondary outcomes included identifying predictors for adherence to prescribing GDMT and one-year mortality. Descriptive statistics and logistic regression analyses were used.

Results

In 460 patients identified, the average age was 61.42 (±11.85) and the majority were male (76.09%). Adherence to prescribing GDMT was achieved in 70.87% of study subjects. The highest prescribing rates were associated with statins (95.22%) and the lowest with ACEIs/ARBs (81.09%). In the multivariable analysis, females and those diagnosed with unstable angina had fewer odds of receiving GDMT (odds ratio [OR]=0.48, 95% confidence interval [CI]=0.30-0.78), and (OR=0.42, CI=0.24-0.75), respectively, while a history of dyslipidemia was associated with higher odds of receiving GDMT. During the one-year follow-up, 23 subjects died in this study, and adherence to GDMT was associated with fewer deaths (OR=0.38, CI=0.16-0.93).

Conclusions

This study shows that there is a pressing need to develop effective strategies to improve compliance with prescribing lifesaving drugs for secondary prevention in subjects with ACS.

## Introduction

Coronary artery disease (CAD) remains the primary cause of death worldwide and in Saudi Arabia despite the availability of highly effective treatments [1-2]. The spectrum of CAD includes stable angina and acute coronary syndrome (ACS), which is the dominant cause of CAD deaths [3]. The use of antiplatelet therapy, high-intensity statin, beta-blockers, and angiotensin-converting enzyme inhibitors (ACEIs) (or angiotensin receptor blockers (ARBs) in patients intolerant to angiotensin-converting enzyme inhibitors) is recommended by several international guidelines for the prevention of secondary events in subjects with ACS [4-8].

Clinical practice guidelines help physicians in clinical decision-making, decrease variability in treatment practices, and improve care [9-11]. Several guidelines exist for the management of ACS, for both ST-elevation myocardial infarction (STEMI) and non-ST-elevation ACS (NST-ACS), such as the National Institute for Health and Care Excellence (NICE) guidelines, the European Society of Cardiology (ESC) guidelines, and the American College of Cardiology/American Heart Association (ACC/AHA) guidelines [4-8]. The guidelines comprise class I recommendations on acute in-hospital pharmacological treatment and prescription of discharge medications [4-8]. Studies show that using the medications recommended by practice guidelines in the inpatient setting and at discharge, together with timely reperfusion therapies, and smoking cessation, in subjects admitted with an ACS, reduces the 30-days mortality rate [12-17].

Several studies have investigated the adherence to guideline-directed medical therapies (GDMT) in patients with ACS and reported a suboptimal adherence [18-22]. Most of these studies focused on assessing the adherence to GDMT in patients with ACS during their hospital stay [19-22]. However, limited data are available on the adherence to GDMT at discharge and its subsequent effect on patient outcomes. Furthermore, little is known about the impact of guideline adherence to GDMT on mortality in ACS patients beyond six months post-discharge.

In Saudi Arabia, patients with ACS are usually slightly different from other populations due to their younger age at presentation and higher prevalence of diabetes as reported by the Saudi Project for the Assessment of Coronary Events registry [23]. One study was recently conducted in the Kingdom, which concluded that subjects who underwent cardiac revascularization surgery are suboptimally discharged on guideline-recommended therapies [18]. However, this study only focused on patients admitted for coronary artery bypass grafting (CABG). Also, the association between adherence to practice guidelines and adverse cardiovascular outcomes was not measured in the study [18].

There is a fundamental need in assessing the adherence to GDMT in ACS patients at discharge and its long-term impact on patients’ outcomes, especially in Saudi Arabia with its high prevalence of cardiovascular disease risk factors. Therefore, this study aims to investigate the impact of adherence to prescribing GDMT at discharge as recommended by the American Heart Association (AHA) practice guidelines for the management of subjects with ACS. We also studied the relationship between various clinical characteristics and adherence to practice guidelines. Furthermore, the association between adherence to GDMT and one-year all-cause mortality was assessed.

## Materials and methods

Study design and setting

A retrospective cohort study was conducted at King Saud University Medical City (KSUMC), a tertiary-care teaching hospital located in Riyadh, Saudi Arabia. Medical records from June 2015 to June 2018 were accessed to collect the study data. Each participant's information was reviewed from the point of hospital admission to one year for the occurrence of the study's primary and secondary outcomes.

Population

Adult patients, 18 years of age and older, consecutively discharged after admission due to an ACS, either STEMI or non-ST elevation ACS (NST-ACS), unstable angina, or NSTEMI, during the study period were included in this cohort. Subjects with a history of CAD but admitted for a reason other than an acute coronary event were excluded from the study. Subjects were followed up for a minimum of one year during the study period. The institutional review board of King Saud University approved the study (institutional review board number: E-18-3271).

Data collection

Information collected from the medical records included age, gender, type of ACS (STMEI or non-ST elevation ACS (NST-ACS), including unstable angina or NSTEMI), admission date, and presence of comorbid conditions (such as cardiovascular disease, lung disease, diabetes mellitus, type of malignancy, liver disease, and renal failure). Information on the medication list, including dosage and frequency, at hospital admission and discharge, allergies or any other contraindications to medications, and one-year mortality were collected.

Study outcomes

The primary study outcome was adherence to prescribing GDMT at discharge based on the AHA practice guidelines recommendations for STEMI and NST-ACS [7-8]. Adherence to treatment guidelines includes prescribing all the following medications at discharge, unless intolerant or contraindicated: aspirin, beta-blockers, and high-intensity statins, which have all have a Class I recommendation. Also, prescribing ACEIs or ARBs, for those patients with left ventricular systolic dysfunction, diabetes mellitus, and/or chronic kidney disease unless the patients had hypotension, hyperkalemia, and worsening renal function, which is a Class I recommendation per the AHA guidelines. To explain this, for example, if a patient did not receive aspirin at discharge, however, that patient had a history of aspirin allergy, or they were intolerant to aspirin, then they would be considered adherent to prescribing aspirin at discharge. This applied to the other GDMT, each with its specific contraindications or intolerance.

Based on the AHA practice guideline, ACEIs or ARBs have a class I recommendation for STEMI and NST-ACS patients with left ventricular ejection fraction (LVEF) less than 0.40 and, in those with hypertension, diabetes mellitus, or stable chronic kidney disease, unless contraindicated [7-8]. The same guidelines provide a class IIb recommendation for ACEIs or ARBs for all patients with STEMI and NST-ACS, regardless of comorbidities [7-8]. Therefore, we have used another definition for adherence in prescribing GDMT at discharge in a sensitivity analysis. For this analysis, adherence to GDMT was defined as prescribing all of the following medications for all patients at discharge, regardless of their comorbid conditions, unless intolerant or contraindicated: aspirin, beta-blockers, high-intensity statins, ACEIs, or ARBs.

The secondary outcome was to identify possible predictors of adherence to GDMT, including patient demographics and clinical characteristics. The third study outcome was to describe the mortality rates, up to one-year follow-up, and study the relationship between all-cause mortality and adherence to prescribing GDMT at hospital discharge.

Statistical analysis

Frequency and percentage analysis described the categorical variables, such as gender, co-existing chronic conditions, and type of ACS. Mean and standard deviation (SD) or median and interquartile range (IQR) were used to describe continuous variables such as age. Chi-square and Fisher's exact tests were used to examine the factors related to adherence to guideline recommendations. Two adherence definitions were adapted, as explained above. The primary analysis was carried out using adherence defined as prescribing all three GDMT (i.e., aspirin, beta-blockers, and high-intensity statins) to all subjects unless contraindicated, and ACEIs and ARBs to those with additional left ventricular systolic dysfunction, diabetes mellitus, and/or chronic kidney disease. A sensitivity analysis was carried out using the second adherence definition. Univariable and multiple regression analyses identified the variables associated with adherence to GDMT, as well as factors associated with one-year all-cause mortality. In the multiple regression models, we controlled for variables that were statistically significant at a p-value ≤0.1 in univariable analyses. All statistical analyses were performed using R version 4.0.3 (R Core Team, Vienna, Austria).

## Results

Study population

A total of 460 patients were included in this study. Table 1 shows the characteristics of the study population. The majority were male (76.09%) with a mean age of 61.42 (±11.85) years. About 68% of the study participants were admitted as STEMI, 19.13% as NSTEMI, and 12.83% as unstable angina. Among the study sample hypertension and diabetes mellitus were the most prevalent health conditions, presenting in 63.04% and 59.77% of the participants, respectively. On the other hand, a previous history of myocardial infarction was only documented in 6.74% of the study subjects. A history of chronic kidney disease and heart failure was present in 1.96% and 1.52% of the study population, respectively.

**Table 1:** Baseline characteristics of the study cohort according to adherence and non-adherence to prescribing GDMT at discharge Abbreviation: ACS, acute coronary syndrome; COPD, chronic obstructive pulmonary disease; GDMT, guideline-directed medical therapy; NSTEMI, non-ST-elevation myocardial infarction; SD, standard deviation; STEM, ST-segment elevation myocardial infarction

Baseline characteristics, n (%)	Overall (n=460)	Adherence to prescribing GDMT (n=326)	Non-adherence to prescribing GDMT (n=134)	p-value
Demographic information				
Female, n (%)	110 (23.91)	67 (20.55)	43 (32.09)	0.012
Age (years), mean ± sd	61.42 ± 11.85	61.25 ± 11.45	61.83 ± 12.81	0.638
Admission ACS type				0.003
STEMI, n (%)	313 (68.04)	227 (69.63)	86 (64.17)	
NSTEMI, n (%)	88 (19.13)	68 (20.86)	20 (14.93)	
Unstable angina, n (%)	59 (12.83)	31 (9.51)	28 (20.90)	
History of comorbid conditions				
Asthma, n (%)	17 (3.70)	11 (3.37)	6 (4.48)	0.775
COPD, n (%)	3 (0.65)	3 (0.92)	0 (0.00)	0.631
Diabetes mellitus, n (%)	275 (59.78)	193 (59.20)	82 (61.19)	0.827
Prior myocardial infarction, n (%)	31 (6.74)	24 (7.36)	7 (5.22)	0.521
Hypertension, n (%)	290 (63.04)	210 (64.42)	80 (59.70)	0.354
Dyslipidaemia, n (%)	92 (20.00)	73 (22.39)	19 (14.18)	0.057
Heart failure, n (%)	7 (1.52)	3 (0.92)	4 (2.99)	0.224
Chronic kidney disease, n (%)	9 (1.96)	4 (1.23)	5 (3.73)	0.167

Adherence to prescribing GDMT at discharge

Overall, 326 (70.87%) subjects who were admitted with an ACS were prescribed the recommended GDMT at the time of discharge (i.e., adherence to prescribing GDMT) (Table 1). The majority of those were admitted due to STEMI (69.63%), and 20.86% were admitted with NSTEMI while only 9.51% were admitted due to unstable angina (Table 1). On the other hand, in the 134 (29.13%) subjects who were not prescribed GDMT as recommended by practice guidelines (i.e., non-adherence to prescribing GDMT) (Table 1). Of those, 64.17% had STEMI at admission, 14.93% had NSTEMI, while unstable angina accounted for 20.90% of admissions (Table 1). In terms of the differences between those who received GDMT at discharge and those who did not, most of the demographic and clinical characteristics were similar, except for the gender, the type of ACS at admission, and a borderline difference in the history of dyslipidemia (Table 1).

**Figure 1:**
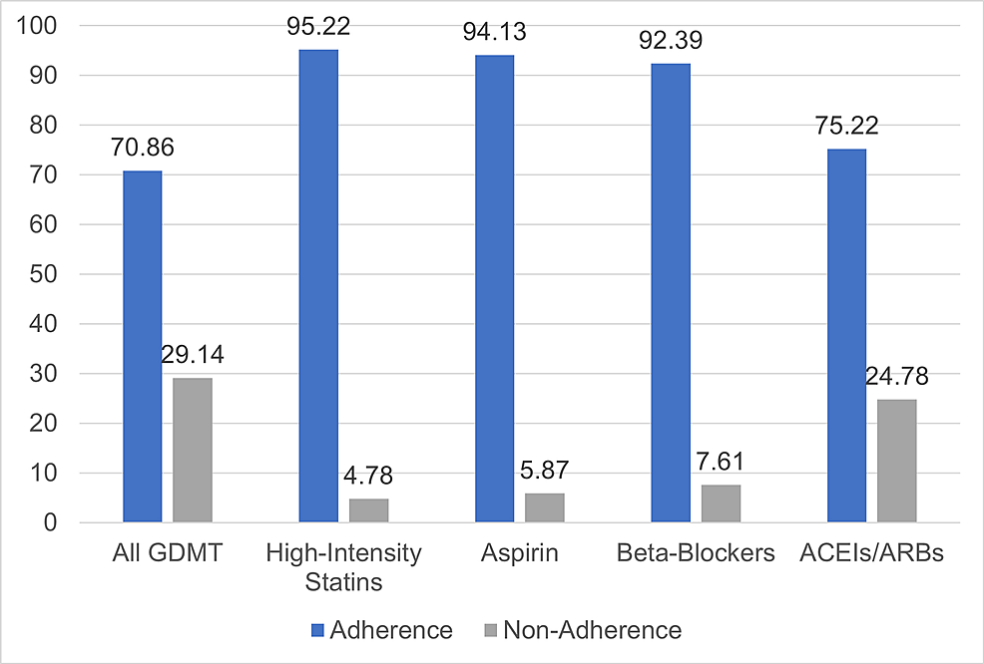
Rate of adherence to prescribing GDMT for each medication Abbreviation: ACEIs, angiotensin-converting enzyme inhibitors; ARBs, angiotensin receptor blockers (ARBs); GDMT, guideline-directed medical therapy.

As seen in Figure 1, none of the GDMTs was prescribed to 100% of the study subjects. Adherence to prescribing high-intensity statins at discharge was achieved in 95.22% of the study subjects, followed by aspirin (94.13%), then beta-blockers (92.39%), and finally ACE/ARBs (81.09%). Using the second definition for adherence to prescribing GDMT, the adherence rate to prescribing ACE/ARBs at discharge dropped to 75.22%, leading to a decrease in overall adherence to prescribing GDMT to 67.83%.

Predictors of adherence to prescribing GDMT at discharge

The results of the univariable and multivariable logistic regression analysis on the outcome of adherence to prescribing GDMT at discharge are available in Table 2. Female subjects had significantly lesser odds of receiving GDMT (OR= 0.55, 95% CI= 0.35, 0.87) in univariable analysis. This was also apparent in multivariable analysis (OR= 0.48, 95% CI= 0.30, 0.78). Furthermore, the admission diagnosis of unstable angina showed a significant reduction in the odds of receiving GDMT in both univariable (OR=0.42, 95% CI=0.24, 0.74) and multivariable analysis (OR=0.42, 95% CI=0.24, 0.75) compared with those admitted with STEMI. After adjusting for possible confounders in the multivariable regression model, patients who had a history of dyslipidemia had significantly twice the odds of receiving GDMT compared to patients without dyslipidemia.

**Table 2:** Predictors of adherence to prescribing GDMT at discharge Abbreviation: ACS, acute coronary syndrome; CI, confidence interval; GDMT, guideline-directed medical therapy; OR, odds ratio; NSTEMI, Non-ST-elevation myocardial infarction; STEM, ST-segment elevation myocardial infarction

Predictors	Univariable analysis			Multivariable analysis		
	OR	95% CI	p-value	OR	95% CI	p-value
Female gender	0.55	0.35, 0.87	0.009	0.48	0.30, 0.78	0.003
Admission ACS type						
STEMI	Reference			Reference		
NSTEMI	1.29	0.75, 2.29	0.400	1.43	0.82, 2.58	0.200
Unstable Angina	0.42	0.24, 0.74	0.003	0.42	0.24, 0.75	0.003
History of dyslipidemia	1.75	1.03, 3.10	0.047	1.84	1.06, 3.33	0.035

One-year all-cause mortality

Of all the study subjects, 23 died during a one-year follow-up. Table 3 shows the demographic and clinical characteristics distributed across the subjects who died at follow-up and those who were alive. The majority of subjects who died during follow-up had a history of diabetes mellitus (69.57%) and hypertension (73.91%). Overall, there was no significant difference in the reported baseline and clinical characteristics between those who died and those alive at the end of the follow-up period, except for age and ACS admission type (Table 3). Furthermore, adherence to prescribing GDMT among the participant who died was only demonstrated in 12 out of the 23 patients (52.17%), (p-value of the difference between the groups=0.074).

**Table 3:** Comparison of baseline data between those who died and those alive at one-year follow-up Abbreviation: ACS, acute coronary syndrome; COPD, chronic obstructive pulmonary disease; GDMT, guideline-directed medical therapy; NSTEMI, non-ST-elevation myocardial infarction; SD, standard deviation; STEM, ST-segment elevation myocardial infarction

Baseline characteristics, n (%)	Alive (n=437)	Dead (n=23)	p-value
Demographic information			
Female, n (%)	104 (23.85)	6 (26.09)	0.999
Age (years), mean ± sd	61.04 ± 11.66	68.70 ± 13.39	0.002
Admission ACS type			0.032
STEMI, n (%)	300 (68.65)	13 (56.52)	
NSTEMI, n (%)	79 (18.08)	9 (39.13)	
Unstable angina, n (%)	58 (13.27)	1 (4.35)	
History of comorbid conditions			
Asthma, n (%)	17 (3.91)	0 (0.00)	0.689
COPD, n (%)	2 (0.46)	1 (4.35)	0.354
Diabetes mellitus, n (%)	259 (59.54)	16 (69.57)	0.460
Prior myocardial infarction, n (%)	29 (6.67)	2 (8.70)	0.999
Hypertension, n (%)	273 (62.76)	17 (73.91)	0.390
Dyslipidaemia, n (%)	89 (20.37)	3 (13.04)	0.556
Heart failure, n (%)	6 (1.38)	1 (4.35)	0.796
Chronic kidney disease, n (%)	8 (1.84)	1 (4.35)	0.941
Adherence to prescribing GDMT, n (%)	314 (71.85)	12 (52.17)	0.074

Predictors of one-year all-cause mortality

The univariable and multivariable regression models for the predictors of mortality are present in Table 4. Age was associated with higher odds of death in both univariable and multivariable analyses. Adherence to prescribing GDMT at discharge was significantly associated with one-year mortality (OR=0.38, CI=0.16, 0.93) in multivariable logistic regression. In addition, patients admitted with NSTEMI had higher odds of death compared to those admitted with STEMI (OR=2.76, CI=1.08, 6.83), even after adjusting for adherence to prescribing GDMT.

**Table 4:** Predictors of one-year mortality Abbreviation: ACS, acute coronary syndrome; CI, confidence interval; GDMT, guideline-directed medical therapy; OR, odds ratio; NSTEMI, non-ST-elevation myocardial infarction; STEM, ST-segment elevation myocardial infarction

Predictors	Univariable analysis			Multivariable analysis		
	OR	95% CI	p-value	OR	95% CI	p-value
Age	1.06	1.02, 1.09	0.003	1.05	1.01, 1.09	0.007
Adherence to prescribing GDMT	0.43	0.18, 1.01	0.048	0.38	0.16, 0.93	0.032
Admission ACS type						
STEMI	Reference			Reference		
NSTEMI	2.63	1.05, 6.32	0.032	2.76	1.08, 6.83	0.029
Unstable angina	0.40	0.02, 2.06	0.400	0.36	0.02, 1.96	0.600

## Discussion

To our knowledge, this is the first study conducted in Saudi Arabia to investigate adherence to prescribing GDMT among patients with ACS at hospital discharge, in addition to identifying predictors of adherence and mortality. Compared to other studies that assessed guidelines adherence among ACS patients, our sample was slightly younger, with more than 60% of the population presenting with chronic comorbidities such as diabetes, dyslipidemia, and hypertension [18,21-24]. Our results proposed that patients with ACS were prescribed suboptimal GDMT at discharge. This includes adherence to prescribing aspirin, statins, ACEIs/ARBs, and beta-blockers. Nearly 29% of the study sample received inadequate GDMT at discharge overall. However, looking at each medication separately, 4.78% of eligible ASC patients did not receive statin therapy at discharge while 5.87% were not prescribed aspirin and 7.61% were not given beta-blockers at discharge. Notably, the highest non-adherent percentage was for prescribing ACEIs/ARBs (18.91%), which is recommended by practice guidelines to subjects with ACS, in addition to having one of the following: LVEF less than 0.40, hypertension, diabetes mellitus, or stable chronic kidney disease.

The findings observed here are consistent with other published studies in the field, especially in the suboptimal adherence with ACEIs/ARBs [19,21-24]. In an observational analysis of hospital care in 350 academic and nonacademic centers in the United States, which consisted of 64,775 patients, overall adherence to ACS guidelines recommendations was achieved in 74% of subjects only [21]. When looking at each medication class separately, a study in Vietnam reported similar adherence rates in prescribing ACEs/ARBs at discharge to our study (89%), however, the same study reported lower adherence to prescribing beta-blockers at discharge than that observed here [20]. In Saudi Arabia, a study conducted on patients discharged from the hospital after CABG showed suboptimal adherence to prescribing GDMT at discharge [19]. For instance, the study investigators found the rate of adherence to prescribing aspirin to be 91%, beta-blockers at 81%, and statin therapy at 84% [19]. Notably, like our findings, the study reports the lowest adherence rates in prescribing ACEIs/ARBs at discharge, which was about 70% [19].

In this study, we highlighted the most common predictors for adherence to prescribing GDMT. History of dyslipidemia was associated with higher odds of receiving GDMT at discharge, which is reflected in the high adherence rates to prescribing high-intensity statins. On the other hand, female subjects and those with unstable angina had lower odds of receiving GDMT. Globally, gender differences exist in subjects admitted with an ACS with respect to both outcomes and treatments [25-26]. Studies vary considerably in the gender differences observed in treatments given at discharge for subjects with ACS [26-32]. Previous reports have shown that females are less likely to receive GDMT in the acute treatment period but not at hospital discharge [26-32]. Various studies indicate that a substantial proportion of patients with non-ST elevation ACS (NST-ACS), i.e., those with unstable angina and NSTEMI, do not receive care according to guidelines recommendations [33-34]. Results from systematic literature reviews show suboptimal guideline adherence in the management of NST-ACS, with an overall 25.0% of patients not receiving appropriate pharmacological treatment [35-36]. These results align with our study that showed lower adherence rates in subjects with unstable angina, a subtype of NST-ACS.

In this study, about 5% of study participants have died during the one-year follow-up after hospital discharge. This mortality rate is inconsistent with the average mortality rates observed in subjects with ACS [37-39]. However, more recent data show similar mortality rates as observed here; this could be reflected by the advances in care and management of subjects with ACS [40]. When looking at possible predictors for mortality, receiving GDMT was associated with significantly lower odds of death [21,41-42]. For example, a 29% reduction in major adverse outcomes at six months after discharge was found for patients of the guideline adherence group compared with the non-adherence group [21]. Also, studies support that adherence to secondary prevention therapy in patients with STEMI is independently associated with lower one-year mortality rates [41-42]. Unfortunately, studies show that if GDMT were not given at discharge, they will rarely be re-introduced later in care, suggesting a further negative impact on mortality and adverse cardiovascular events [41]. Other predictors for mortality were observed here, including age (i.e., an increase in age was associated with higher odds of death) and being diagnosed with NSTEMI at admission. This is consistent with other studies showing that patients with NSTEMI have a higher long-term risk of myocardial infarction and/or death as compared with STEMI patients [43-45].

The study here highlights the need for further steps to improve care and ensure optimal adherence to GDMT in subjects with ACS, especially at hospital discharge. One suggestion would be to add an order alert for subjects with ACS before discharge, and this can be implemented in electronic health records. Pharmacists can take a lead in developing standardized prescription orders for discharge medications in subjects who are admitted with an ACS. Furthermore, at the first clinic visit after discharge, an additional alert system can be added, which may help ensure starting all recommended therapies if the patient was not already on them at discharge. It is also important to note here that we found several factors that are more likely to predict poor adherence to GDMT at discharge. Most importantly, treatment disparities between genders were observed here. Gender bias in the clinical management of cardiovascular disease has been previously documented [46-48]. Despite these apparent disparities, in women’s health, there is still a lack of patient-centered care for women. Furthermore, a scoping review on gender bias in healthcare systems, found that only a few studies have described and evaluated interventions aimed to tackle this bias [49]. The review found that clinical decision-support guidelines and standardized protocols may reduce variability in healthcare and were not specifically designed to reduce gender bias [49]. One study even reports that when a discharge tool is implemented in a healthcare system, this tool was less used in women after acute myocardial infarction than in men [50]. Possibly, multiple interventions or changes in the healthcare system can decrease disparities between genders in medical care in general and for cardiovascular disease specifically. One aspect to focus on would be the education of healthcare professionals on gender bias in the clinical management of subjects with ACS. Furthermore, reforms aimed to include gender aspects can be included in the curricula of medical schools and in health research in order to advance healthcare quality for all genders [51].

Although this study is one of the few studies that investigate the association between adherence to GDTM in ACS patients and one-year mortality, specifically in Saudi Arabia, there are some limitations. First, we only collected single antiplatelet use without investigating dual antiplatelet therapy due to the absence of data in some patients. Second, we were unable to obtain PCI history for some of the study subjects or fibrinolytic use at admission. Third, this study is a single-center retrospective cohort study, which might affect the generalizability of our findings. Forth, mortality data were only obtained from medical records without linkage to death records, which might underestimate the mortality rate observed. Fifth, the definitions we used for guideline adherence might differ from one guideline to another. Finally, we were unable to perform a time-to-death analysis, as the timing of death was not captured in our dataset.

## Conclusions

This is the first study in the region to investigate adherence to prescribing GDMT among patients with ACS at hospital discharge and to assess the impact of this adherence on one-year mortality. The adherence rate to prescribing GDMT at discharge was suboptimal. This includes adherence to prescribing aspirin, statins, ACEIs/ARBs, and beta-blockers. About a third of study participants did not receive adequate GDMT at discharge. Here, a history of dyslipidemia was associated with higher odds of receiving GDMT at discharge while female subjects and those with unstable angina had lower odds of receiving GDMT. This highlights the possible gender differences in hospital care in subjects with ACS. This study also supports the fact that receiving GDMT at discharge can impact mortality. As shown here, patients who received GDMT had significantly lower odds of death at one-year follow-up. Therefore, there is a pressing need to improve care in subjects admitted with ACS, with specific emphasis on female patients.
